# Attitudes towards the role of sports dentistry in high-performance teams: A panel discussion

**DOI:** 10.17159/2078-516X/2025/v37i1a24061

**Published:** 2025-10-15

**Authors:** JP Ganda, L Pillay, C Plaatjies, B Ganda, U Mohammed, J Haughey

**Affiliations:** 1 Wits Sport and Health (WiSH), School of Clinical Medicine, Faculty of Health Sciences, University of the Witwatersrand, Johannesburg-Braamfontein, South Africa; 2 SportsMed Alliance (Pty) Ltd, Cape Town, South Africa; 3 Section Sports Medicine and SEMLI, Faculty of Health Sciences, University of Pretoria, South Africa; 4 Eastern Province Sports & Wellness Institute, Gqeberha (Port Elizabeth), South Africa; 5 Ganda Dental, Roodepoort, Gauteng, South Africa; 6 SmilePlus Dental Care & Implant Centre, Edinburgh, United Kingdom; 7 Sports Dentistry Department, UCL Eastman Dental Institute, London, United Kingdom; 8 Emerald Dental, Dublin, Ireland

**Keywords:** oral health, mouthguards, multi-disciplinary care

## Abstract

Sports dentistry is an emerging area within Sports Medicine that focuses on the prevention, diagnosis and management of oral pathologies in the athletic population. It is not yet a formally recognised subspecialty; however, it is seeking to gain wider acceptance within the context of sports medicine. Oral health is increasingly being linked to overall athlete well-being and performance. The role of the sports dentist encompasses a broad range of oral pathologies that may present in athletes, including oral trauma, periodontal disease, and temporomandibular disorders. Within a high-performance setting, the role of the sports dentist may become integral in the prevention, management and reduction of time-loss due to avoidable dental pathologies. In South Africa, the establishment of the South African Sports Dentistry Association highlights the growing momentum to formalise the field and embed it within the broader high-performance network. Sports dentistry is an evolving field with the potential to enhance player welfare, safety and longevity.

Sports dentistry is a branch of dentistry that focuses on the prevention and treatment of pathologies and injuries affecting the oral cavity and the stomatognathic system.^[1]^ This falls within the realm of sports medicine, which prioritises a multidisciplinary approach to the prevention, aetiology, and treatment of athletes’ diseases. The primary goal is to reduce injury risk and facilitate a faster return to play, with a focus on the overall health and well-being of the athlete.^[2]^ The World Health Organisation (WHO) states that oral health is an integral part of health, wellness and quality of life, and is a fundamental human right.^[3]^

In 2019, the European Association for Sports Dentistry and the Academy for Sports Dentistry developed a consensus statement to promote the role of sports dentists in the broader Sports Medicine community. The priority was to highlight the key roles that sports dentists could fulfil in the broader sports medicine multidisciplinary team and their role in the prevention and treatment of caries, dental erosion, periodontal disease, occlusion/temporomandibular joint disorders (TMD), and finally, the role of the sports dentist in fabricating custom-made, properly fitted mouthguards.^[2]^

Sports dentistry is an emerging field that is gaining significant traction within high-performance sport. Its integration into the broader high-performance medical and performance ecosystem has become increasingly recognised, with a focus on both preventative and acute care.

Key areas of emphasis include pre-season dental screening to identify and address underlying pathology, as well as in-season integration for the fabrication of custom-made mouthguards and the management of dental-related trauma.^[4,5]^ These practices are now being prioritised to ensure athlete safety, optimise performance, and reduce time lost due to avoidable dental issues.

In the South African context, the recent establishment of the South African Sports Dentistry Association (SASDA) reflects this growing momentum. SASDA aims to strengthen the role of sports dentistry, broaden its professional network, and ensure that dental care becomes a recognised and essential component of athlete management in the country.

To gain a better understanding of the role of a sports dentist in a high-performance environment, an expert panel of medical professionals with diverse backgrounds was asked a series of questions. The discussion was facilitated by Dr Janesh Ganda (Sports and Exercise Medicine Physician and Team Doctor for the South African 7s Rugby Team). The panel consisted of Dr Lervasen Pillay (Sports and Exercise Medicine Physician), Dr Clement Plaatjies (Sports and Exercise Medicine Physician and Junior Springbok Team physician), Dr Bhavik Ganda (Dentist), Dr Umair Mohamed (Sports Dentist) and Dr John Haughey (Sports Dentist).

## 1. How do you see the role of the sports dentist in the sports medicine multidisciplinary team?

**Lervasen Pillay:** As part of the Periodic Health Evaluation (PHE)^[5,6]^, sports dentists become involved when issues are identified, whether acute or chronic. Their role includes assisting with custom mouthguards^[4]^ and managing broader dental aspects not directly related to sport, such as TMD or braces, all of which can influence an athlete’s overall well-being.

**Umair Mohammed:** A sports dentist should be regarded as a key member of the sports medicine team, contributing to both the athlete’s health and performance. Their expertise not only supports dental care but also helps identify potential systemic issues that may present orally through routine examinations and screenings. By integrating their knowledge with the broader multi-disciplinary team, overall athlete care and treatment can be optimised. Importantly, many sports dentists also hold, or are encouraged to pursue, advanced pre-hospital care training, enabling them to provide additional support on the sidelines or on the field of play. Their role, therefore, extends beyond managing acute oro-facial trauma to offering a broader scope of care in the high-performance environment.

**Clement Plaatjies**: In collision sports, the role of a sports dentist is particularly important in both prevention and acute management. A key aspect of their contribution is ensuring players have access to well-fitting, protective mouthguards, which are central to safety, especially with the growing use of instrumented mouthguards. Collaboration between sports physicians and sports dentists is vital, as physicians generally have limited expertise in dentistry, and knowledge transfer helps ensure continuity of care. This partnership can extend beyond contact sports; for example, in endurance athletes, where welfare considerations include the risk of dentine dehydration due to mouth breathing and the use of carbohydrate-rich fluids^[6,7]^, the input of a sports dentist can be crucial.

**Bhavik Ganda:** The sports dentist is often an under-recognised member of the sports medicine team, yet plays a vital role that extends beyond dental trauma management. Their responsibilities include preventive care through regular screenings to detect caries, periodontal disease, malocclusion, and bruxism (teeth grinding), as well as managing injuries related to oro-facial trauma, TMD, and providing custom-fitted mouthguards that enhance both protection and breathing efficiency. ^[8]^ They also contribute to performance optimisation by addressing occlusal or jaw-related postural issues that may impact balance and proprioception, while educating athletes and staff on the crucial link between oral health and systemic wellbeing. Ideally, sports dentists work collaboratively with physicians, physiotherapists, nutritionists, and the wider multidisciplinary team to ensure comprehensive, athlete-centred care.^[2]^

**John Haughey:** A sports dentist’s inclusion in the multidisciplinary sports medicine team provides enhanced support for athletes. Their role has both vital and complementary components. The sports dentist provides vital support in areas not covered by other supporting professionals, including oral health, oral trauma and oral function. These areas can impact the athlete’s performance and overall health. Complementary components relate to areas that the sports dentist can support that are not specific to dentistry. Providing this complementary support allows the rest of the sports medicine team to feel supported and can prioritise their areas of expertise. Examples of this include providing support with the removal of an athlete from the field of play or providing suturing support to an athlete, allowing other team members to treat other athletes in areas outside a sports dentist’s scope of practice.

## 2. In your experience, what key dental-related issues do Sports Physicians often overlook, and conversely, what sports medicine aspects do dentists tend to neglect? How can we better close these knowledge gaps without expecting everyone to become experts in both fields?

**Lervasen Pillay:** The role of the sports dentist also extends to addressing chronic issues such as TMD, ensuring players have properly fitted mouthguards, and maintaining overall dental health. As part of the PHE and through ongoing player discussions, they can identify concerns such as sensitive teeth, which are often linked to the high intake of carbohydrate-rich drinks in sport.^[6,7]^ Their input includes practical advice such as recommending appropriate toothpastes and preventive strategies to protect long-term oral health.

**Umair Mohammed:** Oral health in athletes should be assessed with the same rigour as any other aspect of a medical evaluation. From a dental perspective, athletes must undergo screening at least once a year, typically in the off-season, to categorise their oral health risk. A practical system is to classify athletes into three groups:

*Green*, meaning dentally stable and low risk of requiring treatment in-season;
*Amber*, indicating moderate risk where treatment is advised to prevent escalation;
*Red*, highlighting high risk, where urgent care is necessary due to the likelihood of oral health issues impacting training or performance.^[9]^

This approach is essential for both short- and long-term success, as it helps identify and address common pathologies such as gum disease, dental decay, partially erupted wisdom teeth, or abscesses. Given that oral health directly influences an athlete’s nutrition and overall function, it must be managed with the same level of attention and priority as any other bodily system.^[6]^

**Clement Plaatjies**: Basic dental hygiene is often overlooked in athlete care, despite clear links between dental disease and serious conditions, such as infective endocarditis. Factors such as mouth-breathing, high sugar intake, and conditions like sleep apnoea place athletes at increased risk of dental decline.^[4,10]^ These gaps highlight both limited awareness and a lack of research within sports medicine, highlighting the need for closer collaboration with sports dentists to improve care and expand knowledge in this field.

**Bhavik Ganda:** Sports physicians often underestimate the impact of oral health on systemic performance, with conditions such as dental caries, infection, periodontal inflammation, bruxism, and TMD potentially manifesting as headaches, fatigue, or musculoskeletal pain that may be misdiagnosed or overlooked. Conversely, dentists may not fully consider the effects of training loads, injury or concussion history, or posture-related dysfunctions. Bridging this gap does not require practitioners to be experts in every field; rather, it relies on shared education, clear referral pathways, and collaborative case discussions. By recognising overlapping symptoms and maintaining open communication, sports physicians and sports dentists can better address crossover conditions and ultimately improve athlete health and performance outcomes.

**John Haughey:** Historically, in sports, dental support has mainly been provided for oral trauma, either in the treatment of sporting dental injuries or in their prevention, such as the provision of custom-fitted mouthguards. Sports physicians, therefore, generally see provision of oral trauma support as the sole role of a sports dentist. The impact of oral health and oral function on athletic performance and overall well-being is often overlooked. Likewise, sports dentists often see the sports physician’s role to treat athletic injuries. The athlete’s overall well-being and the role the sports physician plays in ensuring the athlete has good health are areas where the sports dentist is less aware. Extensive research demonstrates the connection between oral health and systemic health, highlighting the importance of establishing a close working relationship between the sports dentist and sports physician to provide athletes with enhanced support.

## 3. What challenges are faced in gaining acceptance for sports dentistry as part of the broader multidisciplinary team?

**Lervasen Pillay:** A major challenge is cost. From both the player’s and administrator’s perspective, dental care is often not seen as a priority unless it is directly performance-enhancing. This is further complicated by the fact that many athletes are not on medical schemes, making access and affordability significant barriers to routine dental screening and care.

**Umair Mohammed:** There is a common misconception that sports dentists “just do teeth” or “only provide mouthguards.” In reality, their role extends far beyond this, particularly in the pitch-side or field-of-play environment. Sports dentists often have additional training in prehospital care, allowing them to contribute meaningfully to the management of oro-facial trauma, including lacerations, displacements, and fractures, while providing up-to-date trauma management. Their skill set goes well beyond the dental clinic, and having a sports dentist as part of the team adds both value and reassurance, an extra pair of hands and eyes that ultimately benefits athlete care.

**Clement Plaatjies**: A key question is whether sports dentistry has a large enough footprint within sports medicine to stand alone as a distinct subspecialty. Many of the conditions encountered in athletes overlap with general trauma and routine dental care, with only minor adaptations for sport-specific needs. This raises the challenge of relevance - could a general dentist provide much of the same care as a sports dentist? Possibly, and this is part of the ongoing debate around the unique value and role of sports dentistry in the high-performance setting.

**Bhavik Ganda:** Sports dentistry often struggles for full acceptance despite its relevance to athlete health and performance. A key barrier is the limited appreciation of the systemic and performance implications of oral health among coaches, managers, and even some medical colleagues. Funding priorities typically favour more established disciplines such as physiotherapy or sports science, while minor or chronic dental issues are often overlooked unless trauma occurs. Although evidence linking dental interventions to performance gains is emerging, large-scale studies remain limited. Overcoming these challenges requires ongoing education, sharing case-based evidence, and incorporating oral health screenings into standard medical protocols, emphasising that dental care is essential in athlete management.

**John Haughey:** The main challenges in gaining acceptance of sports dentistry as part of the sports medicine multidisciplinary team are knowledge, awareness, and cost. Knowing all the vital and complementary support roles a sports dentist can provide enables other team members to understand the value of having a sports dentist. Awareness of the needs that athletes have for sports dentistry support helps validate the decision to include a sports dentist in the team. The cost of adding another team member can be a barrier to providing sports dentistry support to athletes. Being creative with the package provided to the sports dentist can offload some of the financial burden and overcome this challenge.

## 4. When an athlete presents with jaw pain, headaches, or balance problems, how do you decide whether the underlying cause is dental or related to sports medicine? What signs do you believe should be more frequently recognised and referred by professionals in the other speciality?

**Lervasen Pillay:** Clinical evaluation is essential to exclude conditions that require urgent attention. This includes assessing vertigo with the Epley’s manoeuvre, conducting Ear, Nose and Throat (ENT) and cardiac examinations to rule out complications such as murmurs caused by subacute bacterial endocarditis (SBE), and considering systemic causes in patients presenting with chronic fatigue, loose teeth, or worsening sensitivity. Broader medical conditions, such as thyroid disease or autoimmune disorders, should also be ruled out before attributing symptoms solely to dental causes.

**Umair Mohammed:** Taking a thorough history is vital, particularly to identify any dental or tooth-related signs or symptoms that may have preceded the presenting pain. A comprehensive intra-oral assessment, supported by appropriate special investigations, helps to rule out dental pathology or referred pain from oral conditions. Once this is excluded, examination of the muscles of mastication and surrounding structures becomes key. Common TMJ or headache-related features include clicking on opening or closing, restricted or deviated mouth opening, and persistent or sharp episodic pain. Importantly, the majority of TMD and headache cases (around 95%) can be managed non-surgically with measures such as anti-inflammatory medication, heat therapy, dietary adjustments, physiotherapy, or the use of a dual-laminate nightguard or splint. ^[7]^ In rarer cases, neuralgia-type pain may be identified through simple intra- and extra-oral testing, an area where sports dentists can add considerable value.

**Clement Plaatjies:** My approach would be to start locally and then move outward when making a diagnosis. In collision sports, these symptoms are common, and in the absence of concussive features, I would first look for a medical explanation. If none can be found, then a dental cause would be considered, making dentistry more of a diagnosis of exclusion unless there is an obvious concern. Conversely, a dentist is likely to begin by seeking a dental cause for the presentation, which highlights the importance of their awareness of medical differentials to avoid partial or missed diagnoses.

**Bhavik Ganda:** Jaw pain, headaches, or jaw disturbances in athletes may stem from medical, dental, or overlapping causes. Clicking, locking, or pain on chewing can indicate TMD or occlusal problems, while musculoskeletal or neurological factors may arise from posture or neck strain. Sports physicians should consider referring patients to a dentist for persistent headaches, bruxism, or a suspected dental infection. At the same time, dentists should refer when symptoms suggest a concussion, systemic fatigue, or non-dental origins. Clear referral pathways and collaboration between professions can reduce misdiagnosis, prevent chronic issues, and deliver more targeted care to enhance athlete performance and wellbeing.

**John Haughey:** A thorough assessment is necessary initially to help determine the cause and severity of the issues. In scenarios where jaw pain presents, a dental examination should always be conducted in conjunction with a sports medicine examination to rule out any dental contribution to the symptoms. Dental abscesses, gum infections, and erupting wisdom teeth are among the issues that can cause athletes to experience jaw pain. If headaches or balance issues present without jaw pain, then, following a sports medicine consultation, a full dental examination should be conducted to assess for any contribution from the athlete’s oral health. Issues like dental abscesses, TMD and misaligned bite conditions can contribute to headaches and balance issues.

## 5. Suppose we could completely rethink how sports organisations handle dental health, disregarding current constraints such as budget, tradition, or logistics. What would the ideal integration of dental care into sports medicine look like? How would we measure success?

**Lervasen Pillay:** Ideally, sports dentists should be involved in full screenings during the PHE and remain available throughout the season for acute care. Few things are more disruptive than an abscess.^[6]^ Regular, repeated dental health screenings, supported by player feedback, are also important to ensure ongoing monitoring and early intervention.

**Umair Mohammed:** The ideal scenario would be for each athlete or sporting organisation to provide sports dentists with dedicated time each year to conduct initial screenings. This would allow athletes to be categorised into oral health risk groups, as previously outlined. While basic screenings can be conducted at training grounds for convenience, more comprehensive assessments are best carried out in a clinical setting, where dental radiography and specialised tests are available. Success would be defined as athletes entering the competitive season without active oral pathology. Where issues do arise, they should be managed promptly and appropriately to ensure they do not disrupt the athlete’s health and performance.

**Clement Plaatjies:** I am not yet convinced that sports dentistry should exist as a standalone subspecialty. However, every athlete should undergo at least an annual dental screening as part of their pre-participation assessment. This would not only allow early detection of issues but also establish a baseline from which changes can be monitored and documented. Significant research is needed to identify dental concerns that occur more frequently in athletes than in the general population. Building a database of such “sports dental pathologies” would allow the development of tailored diagnostic, management, and preventative protocols. In doing so, Sports dentistry could evolve into a recognised subspecialty, functioning with similar structures and metrics to Sports and Exercise Medicine in South Africa.

**Bhavik Ganda:** In an ideal sports medicine framework, oral health would be fully integrated through preseason screenings, routine follow-ups, and inclusion in concussion and facial injury protocols. A sports dentist would collaborate with physicians, physiotherapists, dieticians, and psychologists via shared health records, while also providing custom-fitted mouthguards to enhance protection and compliance. Athlete and staff education on oral–systemic links should be part of wellness programs.

**John Haughey:** The goal of sports dentistry support is to ensure that the athlete’s oral health and oral function do not negatively impact their athletic performance. This includes preventing oral trauma and immediate treatment of oral trauma. To achieve these goals, suggested sports dentistry support includes: 1) Preseason screening to assess that the athlete is dentally fit and there will be no negative oral health or oral function impacts on their performance. Dental treatment to enable the athlete to become dentally fit if needed. Provision of a custom-fitted mouthguard to protect against sporting oral trauma, an instrumented Mouth Guard (iMG) functions and jaw alignment. Dental sleep medicine assessment and provision of a dental sleep appliance if indicated, 2) in-season field of play support to provide immediate oral trauma support, 3) ongoing education on good oral hygiene and provision of oral hygiene products to ensure the athletes have good oral health home care and regular dental hygiene provision to help ensure good oral health and, 4) aesthetic treatments as needed to ensure the athlete has a positive body image of themselves.

Success is measured by the absence of negative impacts on athletic performance due to the athlete’s oral health. Reviewing injury prevalence, incidence, return-to-play time (time lost), and training availability, as well as the athlete’s performance before and after sports dentistry input, can help provide success measures.

## Conclusion

Sports dentistry is an emerging field which is gaining momentum in high-performance teams. Its integration encompasses the facilitation of pre-season oral health screening, acute injury management of dental origin and management of oral health pathologies that are often not well recognised by team physicians. These can result in missed training days and negatively impact the team’s performance. The provision of mouthguards in a collision sport like rugby union is also an ideal setting in which sports dentists can be involved—the fabrication and fitting of custom-made mouthguards, which are both essential to an athlete’s performance and well-being. Despite concerns around the footprint of sports dentistry, as awareness and research into this field increase, the contribution of sports dentists will become more apparent in the success of high-performance teams.
